# 0.1% octenidine versus 0.2% chlorhexidine mouthwashes for plaque-induced gingivitis: A triple-blinded randomized controlled trial

**DOI:** 10.6026/973206300223405

**Published:** 2026-06-30

**Authors:** Sharvari Pokale, Sneh Agrawal, Prajakta Rao, Pranita Dalave, Vivek Sharma, Sanpreet Singh Sachdev

**Affiliations:** 1 Department of Periodontology, Bharati Vidyapeeth (Deemed to be University) Dental College and Hospital, Navi Mumbai, India

**Keywords:** Plaque-induced gingivitis, octenidine dihydrochloride, chlorhexidine digluconate, antimicrobial mouthrinse, gingival inflammation

## Abstract

Plaque-induced gingivitis is commonly managed by mechanical plaque control, but adjunctive mouthrinses may improve outcomes. Therefore, it is of interest to compare 0.1% octenidine, 0.2% chlorhexidine and placebo in 66 patients with plaque-induced gingivitis treated with scaling and root planing. Gingival index, plaque index, stain index and colony-forming units were recorded at baseline, 7 days and 15 days. Both octenidine and chlorhexidine produced significant reductions in gingival inflammation, plaque and microbial load compared with placebo, while chlorhexidine caused significantly greater staining. Thus, 0.1% octenidine showed efficacy comparable to 0.2% chlorhexidine with better cosmetic acceptability, supporting its use as an alternative adjunct in plaque-induced gingivitis.

## Background:

Gingivitis remains one of the most prevalent oral diseases globally and serves as an early stage in the continuum toward periodontitis. It is initiated primarily by the accumulation of microbial plaque biofilm on tooth surfaces and gingival margins, which elicits an inflammatory host response. If unmanaged, gingivitis can progress to irreversible attachment loss, alveolar bone destruction and eventual tooth loss, thereby posing a major public health concern [1]. The control of plaque biofilm primarily relies on the patient's motivation and effective mechanical cleaning through tooth brushing [2]. However, certain areas of the teeth are sometimes inaccessible to toothbrushes, especially if malocclusion is present. Variations in brushing technique, manual dexterity and compliance often compromise the efficacy of mechanical plaque control alone, necessitating the adjunctive use of chemical antiplaque agents [2, 3]. Among chemical agents, chlorhexidine digluconate (CHX) remains the most widely studied and clinically accepted "gold standard" in reducing gingival inflammation and plaque accumulation [4]. Its cationic bisbiguanide structure confers broad- spectrum antimicrobial activity and high substantivity, allowing sustained release on oral surfaces. Nevertheless, its long-term use is limited by undesirable side effects. These include brownish staining of teeth and tongue, altered taste sensation, parotid enlargement, and occasional mucosal irritation [5]. These drawbacks underscore the need for alternative antimicrobial mouthrinses that achieve comparable plaque control with improved patient acceptance. Octenidine dihydrochloride (OCT) is a newer bispyridine compound with a strong cationic charge that interacts with bacterial cell membranes, leading to leakage of cytoplasmic components and cell death [6]. Unlike CHX, OCT demonstrates minimal systemic absorption, rapid bactericidal action, and excellent safety even in mucosal applications. It exhibits potent activity against both Gram-positive and Gram-negative bacteria, fungi, and some viruses, making it a promising candidate for oral antisepsis [7]. Preliminary *in vitro* and *in vivo* studies have reported its efficacy in reducing microbial load without inducing significant staining or taste disturbances. These suggest a favorable benefit-risk profile for long-term use [6, 7, 8]. Given these advantages, further evaluation of OCT compared with CHX is warranted to determine its clinical applicability for routine plaque control. Very few studies have compared the anti- plaque effectiveness of Octenidine and Chlorhexidine mouthwashes to date. Therefore, it is of interest to assess and compare the clinical and microbiological efficacy of 0.1% octenidine dihydrochloride and 0.2% chlorhexidine digluconate mouthrinses as adjuncts to mechanical therapy in managing plaque-induced gingivitis.

## Materials and Methods:

The present triple-blinded, randomized, placebo-controlled clinical trial was conducted from October 2023 to November 2024 in a dental institute in Navi Mumbai. The study protocol was approved by the Institutional Ethics Committee (Biomedical Ethics Committee, Reference No.: IEC332072022, dated 09/08/2023). All procedures were conducted in accordance with the Declaration of Helsinki and written informed consent was obtained from each participant before enrolment.

## Sample selection and eligibility criteria:

A total of n=66 participants aged 18 to 30 years were recruited from the institutional Department of Periodontics. All participants were systemically healthy and presented with chronic plaque-induced gingivitis, characterized by gingival inflammation without attachment loss and a baseline Silness and Löe plaque index score of less than 1. Inclusion criteria comprised willingness to comply with study procedures, presence of a minimum of 20 natural teeth and absence of any systemic condition that could influence periodontal status. Patients with a history of antibiotic or anti-inflammatory therapy within the previous three months, known hypersensitivity to any component of the mouthrinses, tobacco, or betel-nut use were excluded. Patients with systemic disorders and pregnant and lactating women were excluded from the study. Participants who failed to adhere to instructions or discontinued use of the assigned rinse were withdrawn from the study.

## Randomization and blinding:

Eligible participants were randomly allocated into three groups (n=22 per group) using the chit-in-box method to ensure simple randomization. Group A received scaling and root planing (SRP) followed by 0.1% OCT mouthrinse (Octenoxa®, Glenmark Pharmaceuticals Ltd, Mumbai, India). Group B received SRP followed by 0.2% CHX mouthrinse (Hexidine®, ICPA Health Products Ltd, Mumbai and Ankleshwar, India). Group C received SRP followed by placebo (distilled water). To ensure triple blinding, the participants, clinician and statistician were all unaware of group allocations. Mouthrinses were distributed in identical opaque bottles, coded by an independent investigator (SA) who was not involved in data collection or analysis.

## Clinical procedures and oral hygiene instructions:

All participants underwent professional mechanical plaque removal through scaling and root planing at baseline. They were provided with soft-bristled toothbrushes (Oral-B Cavity Defense, Mumbai, India) and non-fluoridated toothpaste (Botanique Complete Care, Bengaluru, India). The participants were instructed to brush using the modified Bass brushing technique throughout the study period. Participants were asked to rinse with 10 mL of the assigned mouthwash twice daily for 1 minute, approximately 30 minutes after brushing. The participants were also instructed to avoid eating, drinking, or rinsing with water for 30 minutes thereafter. Compliance was monitored through a log maintained by the participants and verified at follow-up visits.

## Clinical assessment:

Clinical parameters were recorded at baseline, 7 days, and 15 days. These included Silness and Löe Plaque Index [9], Modified Gingival Index [10] and Modified Lobene Stain Index [11]. A single calibrated examiner took all measurements to minimize inter-observer variability. Calibration was achieved through repeated assessments of 10 non-study subjects until intra-examiner consistency exceeded 90%.

## Microbiological analysis:

For microbiological evaluation, supragingival plaque samples were collected at a fixed time interval in the mornings to minimize diurnal variation. Participants were instructed to refrain from eating, drinking, or brushing their teeth for at least two hours before sample collection. Plaque samples were transferred into sterile containers containing normal saline and processed within 24 hours. The samples were vortexed to disperse bacterial aggregates, then diluted in series and plated on blood agar (Figure 1 - see PDF). The plates were incubated aerobically at 37°C for 24 hours. The colony-forming units (CFUs) were counted digitally using a colony counter. The results were expressed as CFU/mL of plaque sample.

## Statistical analysis:

Data were compiled and analyzed using IBM SPSS Statistics for Windows, Version 26.0 (IBM Corp., Armonk, NY, USA). Descriptive statistics were expressed as mean ± standard deviation. Normality of data distribution was tested using the Shapiro-Wilk test. As the data were non-parametric, intra-group comparisons across different time intervals were analyzed using Friedman's test, followed by Wilcoxon signed- rank post-hoc analysis. Inter-group comparisons were made using Kruskal-Wallis ANOVA and Mann-Whitney U tests. The significance level was set at p<0.05, and statistical power was maintained at 80% (α=0.05, β=0.20).

## Results:

A total of 66 participants were enrolled, evenly divided into three study groups (n=22 per group), and all completed the study (Figure 2 - see PDF). No participant reported adverse reactions or required discontinuation and overall compliance with the assigned mouthwash regimen was high. Baseline comparison confirmed that the groups were statistically comparable for GI, PI, CFU counts, stain intensity and stain (intensity x area). Only the baseline stain area showed a statistically significant difference among groups. All other clinical and microbiological measures at baseline demonstrated no significant variation, indicating adequate homogeneity before initiating the interventions (Table 1). All three groups demonstrated statistically significant intra-group changes across the 15-day period, as confirmed by the Friedman test (Table 2). For the octenidine intervention, significant differences (p<0.01) were noted across all intervals for the Gingival Index, Plaque Index and CFU counts. Subsequent Wilcoxon pairwise comparisons demonstrated progressive reductions in all three parameters from baseline to Day 7 and from Day 7 to Day 15 (all p<0.01). Likewise, the chlorhexidine group showed statistically significant overall changes in GI, PI and CFU across the three time points in the Friedman test (p<0.01). Pairwise comparisons again revealed significant reductions from baseline to Day 7 and from Day 7 to Day 15 (all p<0.01), indicating consistent improvement following chlorhexidine use. In the placebo group, the Friedman test also indicated significant overall changes in GI, PI and CFU across the study duration (p<0.05 for GI; p<0.01 for PI and CFU). However, Wilcoxon pairwise testing showed a different pattern compared to the active groups. GI reduction from baseline to Day 7 was not statistically significant (p>0.05). However, a significant improvement was observed from baseline to Day 15 and from Day 7 to Day 15 (both p<0.05). PI and CFU showed statistically significant changes across all time intervals (p<0.01), although the direction of change differed. PI showed a mild reduction, while CFU levels fluctuated rather than showing consistent improvement as seen with octenidine and chlorhexidine. Stain index components (intensity, area and intensity x area) also showed significant time-dependent changes in all three groups (p<0.01). Wilcoxon post-hoc comparisons showed significantly greater increases in staining at both Day 7 and Day 15 in the chlorhexidine group compared with baseline (all p<0.01). In contrast, octenidine showed smaller but still significant increases (p<0.01). The placebo group showed statistically significant, but comparatively small, increases in stain indices over time (p<0.01). Collectively, inclusion of the Friedman test results strengthens the intra-group interpretation, confirming that the observed changes over time were not incidental. Octenidine and chlorhexidine demonstrated statistically robust improvements in gingival inflammation, plaque accumulation and microbial load. On the other hand, the placebo group showed limited or inconsistent improvement. Inter-group analysis demonstrated that there were no statistically significant differences (p>0.05) among the three groups at baseline for GI, PI, or CFU (Table 3). By Day 7 and Day 15, however, significant differences were observed for all three clinical and microbiological parameters (p<0.01). Both octenidine and chlorhexidine showed markedly lower GI and PI values than the placebo at both follow-up intervals (p<0.01). The differences between the two active rinses were more limited and reached significance only for PI at Day 7 and Day 15 (p<0.01). For CFU, both active mouthrinses demonstrated significantly lower microbial counts (Figure 3 - see PDF) compared to placebo at both Day 7 (p<0.05) and Day 15 (p<0.01). Pairwise comparisons confirmed significant differences for octenidine versus placebo and chlorhexidine versus placebo on both days. Octenidine and chlorhexidine differed from each other only at Day 7 for CFU (p<0.05), with no significant difference at Day 15 (p>0.05). Inter-group comparisons of stain index parameters revealed no significant difference in baseline stain intensity (p>0.05). The stain area and intensity x area differed significantly among the groups at baseline (p<0.01) (Table 4). By Day 7 and Day 15, all stain parameters demonstrated highly significant differences among the three groups (p<0.01). Pairwise analyses (Table 5) showed that chlorhexidine produced significantly greater staining parameters compared with both octenidine and placebo at both follow-up points (p<0.01). Octenidine also differed significantly from placebo in all stain measures at Day 7 and Day 15 (p<0.01). This indicated moderate staining with octenidine and minimal staining with the placebo. Overall, stain outcomes displayed a consistent hierarchy of chlorhexidine, followed by octenidine and lastly, placebo.

## Discussion:

The present study demonstrated that both 0.1% octenidine dihydrochloride and 0.2% chlorhexidine digluconate produced clear clinical improvements in plaque-induced gingivitis. This was reflected by marked reductions in gingival inflammation, plaque accumulation, and microbial load over 15 days. These findings are consistent with the established antimicrobial profiles of both agents, which act primarily by disrupting bacterial membranes. However, the mechanisms differ in their biophysical details. Octenidine exerts rapid, mechanically driven membrane destabilization. While, chlorhexidine binds strongly to negatively charged bacterial surfaces and alters cellular integrity through cationic interactions [12]. The significant microbiological reductions observed follow this pharmacodynamic rationale and align with multiple earlier studies in which both agents demonstrated potent anti-plaque and anti-gingivitis effects [13, 14]. Comparative performance between the two antiseptics in this study indicates that octenidine achieved antimicrobial and clinical outcomes comparable to chlorhexidine. Similar equivalence has been observed in plaque regrowth model, in which octenidine and chlorhexidine exhibited nearly identical inhibitory effects on plaque formation [15]. The consistency of these findings across different study designs strengthens the interpretation that octenidine is clinically comparable to chlorhexidine in the short term. The antimicrobial behavior observed is also strongly supported by earlier *in vitro* and *in vivo* work. Previous work has demonstrated that octenidine has greater or equal inhibitory activity than chlorhexidine against a range of oral microorganisms. These include streptococci, lactobacilli, and periodontal pathogens [16]. Overall, the findings of the present study, supported by earlier reports, indicate that octenidine achieves better plaque control across diverse microbial environments. It also exhibits faster, more sustained killing kinetics than chlorhexidine. One of the most notable distinctions revealed in this study relates to the staining profiles of the two agents. Chlorhexidine produced substantially higher increases in stain intensity, stain area, and composite stain index, whereas octenidine resulted in markedly milder staining. This pattern mirrors clinical findings reported by Rath et al. [17], that a significantly greater extrinsic discoloration with chlorhexidine. The biochemical basis for this difference is attributable to the strong interaction of CHX with dietary chromogens and its ability to promote Maillard-type browning reactions on pellicle surfaces [18]. Octenidine lacks this strong chromogenic affinity, resulting in a more favorable cosmetic profile, which may enhance patient acceptability and adherence [9, 18]. Recent clinical evidence also supports the present findings. Verma *et al.* reported that 0.1% octenidine improved plaque and gingival scores after SRP in patients with gingivitis and periodontitis [19]. Neha and Basheer also found better plaque, bleeding and probing-depth outcomes with octenidine than chlorhexidine over 90 days [20]. These findings support octenidine as an effective alternative to chlorhexidine. However, comparisons should be made with caution because follow-up durations and measured outcomes differed across studies. While the findings strongly support the clinical utility of both agents, the study is limited by its short duration. This restricts insight into long-term stain progression, potential shifts in microbial resistance, and sustained periodontal benefits. The microbiological assessment used culture-based CFU counts. Although informative, do not provide species-level or biofilm-specific insights that molecular techniques could yield. Additionally, the study did not evaluate patient-reported outcomes such as taste alteration or oral comfort, which are relevant to real-world compliance. Future research should incorporate more extended follow-up periods, larger and more diverse populations, molecular microbial analyses, and broader patient-centered assessments. Evaluating different concentrations and formulations of octenidine is essential. Studying their role in managing periodontitis, peri-implant mucositis, or high-risk caries populations would further clarify their therapeutic value.

## Conclusion:

We show that both 0.1% octenidine and 0.2% chlorhexidine are effective adjuncts to mechanical therapy in managing plaque-induced gingivitis. Octenidine provided clinical and microbiological benefits comparable to those of chlorhexidine while causing less staining. Thus, data shows octenidine as a reliable alternative to chlorhexidine where efficacy and cosmetic acceptability are equally important. Further long-term studies are recommended to evaluate sustained outcomes and patient adherence.

## Figures and Tables

**Table 1:** Mean and standard deviation of the baseline characteristics of the study participants (N=66)

**Parameter**	**Group A (OCT) (n=22)**	**Group B (CHX) (n=22)**	**Group C (Placebo) (n=22)**	**p-value (Kruskal-Wallis)**
Gingival Index (GI)	2.73± 0.985	2.73± 0.935	2.68± 0.894	0.994#
Plaque Index (PI)	1.668± 0.390	1.673± 0.398	1.723± 0.375	0.638#
CFU/mL	634.23±321.19	638.05± 223.01	633.77± 322.07	0.977#
Stain Intensity	0.383± 0.092	0.394± 0.089	0.375± 0.075	0.475#
Stain Area	1.353± 0.107	1.369±0.107	0.917± 0.138	0.000**
Stain I x A	0.580± 0.322	0.541±0.134	0.346± 0.089	0.000**
(#=non-significant,
**=significant at p<0.01)

**Table 2:** Intra-Group Changes in GI, PI and CFU from Baseline to Day 7 and Day 15 (Friedman Test)

**Parameter**	**Time point/ Test statistic**	**Group A (OCT) Mean± SD**	**Group B (CHX) Mean± SD**	**Group C (Placebo) Mean± SD**
Gingival Index (GI)	Baseline	2.73± 0.99	2.73± 0.94	2.68± 0.89
	Day 7	1.18± 0.80	1.45± 0.86	2.77± 1.02
	Day 15	0.45± 0.60	0.68± 0.72	3.09± 0.81
	χ^2^ value	40.43	38.68	8.67
	p-value	0.000**	0.000**	0.013*
Plaque Index (PI)	Baseline	1.668± 0.390	1.673± 0.398	1.723± 0.375
	Day 7	0.959± 0.300	1.305± 0.429	1.868± 0.373
	Day 15	0.582± 0.261	1.109± 0.482	2.195± 0.462
	χ^2^value	44	43.52	38.52
	p-value	0.000**	0.000**	0.000**
CFU (mL)	Baseline	634.23± 321.19	638.05± 223.01	633.77± 322.07
	Day 7	478.77± 241.87	512.86± 199.41	688.59± 291.50
	Day 15	343.68± 188.25	389.86± 181.20	747.68± 280.50
	χ^2^ value	42.09	44	38.27
	p-value	0.000**	0.000**	0.000**
(*p<0.05,
**p<0.01)

**Table 3:** Inter-Group Comparison of GI, PI and CFU at Baseline, Day 7 and Day 15 (Kruskal-Wallis Test)

**Parameter**	**Time point**	**Group A Mean± SD**	**Group B Mean± SD**	**Group C Mean± SD**	** χ^2^ value**	**p-value**
Gingival Index (GI)	Baseline	2.73± 0.99	2.73± 0.94	2.68± 0.89	0.012	0.994#
	Day 7	1.18± 0.80	1.45± 0.86	2.77± 1.02	23.506	0.000**
	Day 15	0.45± 0.60	0.68± 0.72	3.09± 0.81	44.645	0.000**
Plaque Index (PI)	Baseline	1.668± 0.390	1.673± 0.398	1.723± 0.375	0.898	0.638#
	Day 7	0.959± 0.300	1.305± 0.429	1.868± 0.373	32.812	0.000**
	Day 15	0.582± 0.261	1.109± 0.482	2.195± 0.462	46.425	0.000**
CFU (mL)	Baseline	634.23± 321.19	638.05± 223.01	633.77± 322.07	0.047	0.977#
	Day 7	478.77± 241.87	512.86± 199.41	688.59± 291.50	7.134	0.028*
	Day 15	343.68± 188.25	389.86± 181.20	747.68± 280.50	21.298	0.000**
(# Non-significant;
*p<0.05; **p<0.01)

**Table 4:** Inter-Group Comparison of Stain Index Parameters (Intensity, Area, I×A) at Baseline, Day 7 and Day 15 (Kruskal-Wallis Test)

**Parameter**	**Time point**	**Group A Mean± SD**	**Group B Mean± SD**	**Group C Mean± SD**	** χ² value**	**p-value**
Stain Intensity (I)	Baseline	0.383± 0.092	0.394± 0.089	0.375± 0.075	1.489	0.475#
	Day 7	0.430± 0.092	0.571± 0.045	0.386± 0.076	41.77	0.000**
	Day 15	0.449± 0.091	0.780± 0.069	0.391± 0.075	46.38	0.000**
Stain Area (A)	Baseline	1.353± 0.107	1.369± 0.107	0.917± 0.138	43.624	0.000**
	Day 7	1.413± 0.110	1.502± 0.087	0.908± 0.225	46.674	0.000**
	Day 15	1.431± 0.109	1.737± 0.066	0.912± 0.226	57.696	0.000**
Stain Index (I x A)	Baseline	0.580± 0.322	0.541± 0.134	0.346± 0.089	25.077	0.000**
	Day 7	0.610± 0.144	0.814± 0.177	0.354± 0.116	45.177	0.000**
	Day 15	0.645± 0.146	1.356± 0.136	0.360± 0.114	54.421	0.000**
(# non-significant;
** p<0.01)

**Table 5:** Pairwise Inter-Group Comparisons Using Mann-Whitney U Test for GI, PI, CFU and Stain Index Parameters

**Parameter**	**Time point**	**A vs B (p-value)**	**A vs C (p-value)**	**B vs C (p-value)**
Gingival Index (GI)	Baseline	0.960#	0.902#	0.970#
	Day 7	0.321#	0.000**	0.000**
	Day 15	0.288#	0.000**	0.000**
Plaque Index (PI)	Baseline	0.972#	0.376#	0.457#
	Day 7	0.006**	0.000**	0.000**
	Day 15	0.000**	0.000**	0.000**
Microbial Load (CFU)	Baseline	0.981#	0.963#	0.742#
	Day 7	0.597#	0.016*	0.034*
	Day 15	0.372#	0.000**	0.000**
Stain Intensity (I)	Baseline	0.526#	0.466#	0.254#
	Day 7	0.000**	0.038*	0.000**
	Day 15	0.000**	0.010**	0.000**
Stain Area (A)	Baseline	0.347#	0.000**	0.000**
	Day 7	0.007**	0.000**	0.000**
	Day 15	0.000**	0.000**	0.000**
Stain Intensity x Area	Baseline	0.597#	0.000**	0.000**
	Day 7	0.000**	0.000**	0.000**
	Day 15	0.000**	0.000**	0.000**
(# non-significant;
* p<0.05; ** p<0.01)
